# G22A Polymorphism of Adenosine Deaminase and its Association with Biochemical Characteristics of Gestational Diabetes Mellitus in an Iranian Population

**Published:** 2015-03

**Authors:** Mohammad Ali Takhshid, Zinab Zahediannejad, Farzaneh Aboualizadeh, Leili Moezzi, Reza Ranjbaran

**Affiliations:** Diagnostic Laboratory Sciences and Technology Research Center, School of Paramedical Sciences, Shiraz University of Medical Sciences, Shiraz, Iran

**Keywords:** Adenosine deaminase, Gestational diabetes, Single nucleotide polymorphism G22A

## Abstract

Adenosine deaminase (ADA) is an important regulator of insulin action. The single nucleotide polymorphism (SNP) G22A in the ADA gene decreases enzymatic activity of ADA. The aim of this study was to investigate the relationship between the SNP G22A and blood glycemic control, insulin resistance, and obesity of gestational diabetes mellitus (GDM) patients in an Iranian population. SNP G22A was determined in women with GDM (N=70) and healthy pregnant women (control, N=70) using polymerase chain reaction-restriction fragment length polymorphism. Fasting plasma glucose (FPG), hemoglobin A1C (HbA1c), plasma insulin levels and plasma lipids were measured using commercial kits. Homeostasis model of assessment for insulin resistance (HOMA-IR) was calculated. The distribution of genotypes and alleles among GDM patients was similar to that of the control group. FPG and HbA1c were significantly higher in GDM patients with GG genotype compared with GDM patients with GA+AA genotype and non-GDM patients. The frequency of GG genotype was significantly higher in obese GDM patients compared to lean GDM patients. The SNP G22A in the ADA gene was not associated with the risk of GDM in our population. GG genotype was associated with poor glycemic control and obesity in GDM patients.

## Introduction

Gestational diabetes mellitus (GDM) is defined as glucose intolerance that is developed or first recognized during pregnancy.^1^ GDM and type 2 diabetes (T2D) may have common pathogenic mechanisms. Obesity, insulin resistance, and genetic susceptibilities are among the common antecedent of both GDM and T2D.^2^^,^^3^ Adenosine deaminase (ADA; EC 3.5.4.4), a key enzyme in purine metabolism, regulates extracellular and intracellular concentrations of adenosine by irreversible deamination of adenosine into inosine.^4^ The role of ADA has been implicated as an important regulator of insulin action and in the pathogenesis of diabetes mellitus. Increased serum levels of ADA and its correlations with glycemic control have been reported in diabetic patients.^5^^,^^6^ ADA is encoded by the polymorphic ADA gene, which is located on chromosome 20q13.11. One of the commonest single nucleotide polymorphisms (SNP) of ADA gene is the SNP G22A in exon1. This SNP results in the substitution of Asp amino acid (G allele) with Asn (A allele) amino acid in position 8 of the enzyme. This amino acid substitution decreases catalytic activity of ADA. Consequently, individuals with the A allele show reduced ADA enzymatic activity compared to homozygous GG individuals.^7^

The aim of the present study was to investigate the association between SNP G22A in the ADA gene with the risk of GDM. We also assessed relationships between SNP G22A and fasting plasma glucose levels, HbA1c and insulin resistance in pregnant women with GDM. 

## Materials and Methods

A sample of 140 unrelated pregnant Iranian women was enrolled as the study group. The pregnant women were classified as non-diabetic control group (N=70) and having GDM (N=70) according to oral glucose tolerance test (OGTT) results. OGTT was performed according to the American Diabetes Association 2009 criteria, between the 24^th^ and 28^th^ week of gestation.^8^ Maternal BMI (kg/m^ 2 ^) was calculated as the ratio of the weight (kg) to the square of the height (m). The inclusion criteria for the pregnant women with GDM were; newly diagnosed cases and no previous use of oral hypoglycemic agents. The exclusion criteria were the presence of type-1 or type-2 diabetes mellitus and other known major diseases. The study protocol was approved by the Ethics Board of Shiraz University of Medical Sciences, Shiraz, Iran. Informed written consent was obtained from all participants.

Fasting venous maternal blood sample was collected in both groups. Sera were separated immediately and used for biochemical analyses. Fasting plasma glucose (FPG), total cholesterol (TC), triglyceride (TG) and high-density lipoprotein cholesterol (HDL-C), HbA1c and serum insulin levels were measured using commercially available kits. Homeostasis model of assessment for insulin resistance (HOMA-IR) was used for the evaluation of insulin resistance.^9^

**Figure 1:**
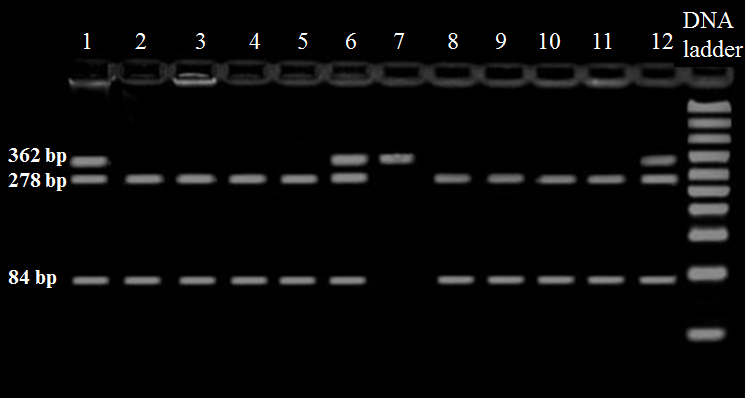
Representative 2.0% agarose gel picture. Gel electrophoresis of digested PCR product by the restriction enzyme *Taq* I. Lane 1, 6, 12-GA genotype, lane 2, 3, 4, 5, 8 ,9 ,10 ,11-GG genotype; lane 7-AA genotype.

Genotyping for detection of SNP G22A polymorphisms of the ADA gene was performed using polymerase chain reaction-restriction fragment length polymorphisms method.^7^ In brief, a DNA fragment (362 bp) containing the SNP G22A was amplified by 5′-ACCGAGCCGGCAGAGACCCAC-3′ as forward primer and 5′ACTTGAC-AGACAGCGAAACTGAGACCCAGA-3′ as reverse primer. PCR products were digested by Taq I restriction enzyme (Fermentas Life Sciences, Lithuania) and separated on 2% agarose gel electrophoresis. The PCR product corresponding to G allele was then cleaved into two fragments; a 278 bp fragment and an 84 bp fragment. The *A* allele was identified by the absence of the *Taq *I restriction site (a 364 bp fragment) (figure 1).

*Statistical Methods*

Results were analyzed using SPSS software version 15.0. Comparisons of different variables between groups were done using Student’s t-test or one way ANOVA. The chi-square test was used to compare frequencies of allele and genotypes. For all comparisons, the statistical significance was defined by a P<0.05. 

## Results

The baseline demographic and clinical characteristics of patients with GDM and controls are shown in table 1. The maternal age, gestational age and BMI of the subjects were similar in the groups. GDM women had significantly higher HOMA-IR (P=0.005), FPG (P=0.002) and HbA1C (P<0.001) than non-GDM subjects. The genotype and allele frequencies in our case-control samples are shown in table 2. All variants are in the Hardy-Weinberg equilibrium. The results suggested that there was no significant association between GDM and the distributions of SNP G22A genotypes and alleles (table 2). Further analysis revealed that the frequency of GA+GG genotypes was also similar between GDM patients and non-GDM subjects (P=0.385 OR (95% CI), 1.46 (0.62-3.5)) (table 3). Table 4 summarizes the mean of various biochemical characteristics in carriers of GG genotype and carriers of A alleles (GA+AA genotypes group). GDM patients with GG genotype had a significantly higher FPG compared to non-GDM subjects with GG genotype and GA+AA genotypes (P=0.002 and P=0.007 respectively). They also had a higher HbA1c when compared to non-GDM subjects with GG genotype and GA+AA genotypes (P<0.001 and P<0.001 respectively). HOMA-IR tended to be higher among GDM subjects with GG genotype, although not statistically different from the non-GDM subjects with GG genotype and GA+AA genotypes (P=0.07 and P=0.085 respectively) (table 4). To examine whether the presence of the SNP G22A was related to BMI, we divided the GDM patients into 3 groups according to pregnancy BMI: ≥25 kg/m^ 2^ (group A), 26-29 kg/m^ 2^ (group B) and *≥*30 kg/m^ 2^ (group C). An increase in frequency of GG genotype was observed with an increase in BMI. The frequency of GG genotype in group C was significantly higher than group A (91% and 64%, respectively P=0.049). In group B, the frequency of the GG genotype (76%) did not differ significantly from that of group A (P=0.168).

**Table 1:** Demographic characteristics and biochemical measurements in the GDM and non-GDM subjects

	**Non-GDM group (n=70)**	**GDM group (n=70)**	**P value**
Maternal age (year)	26.7±5.0	29.4±4.9	0.73
Gestational age (week)	29.6±3.1	29.9±3.4	0.60
BMI (Kg/m^ 2 ^)	28.1±4.4	28.2±4.5	0.94
FPG (mg/dl)	79±11	90±22	0.002*
HbA1C (%)	5.1±0.37	5.8±0.95	<0.001*
Insulin (µg/dl)	17.5±7.6	18.2±11.6	0.67
HOMA-IR	3.4±1.6	4.6±3.1	0.005*
TG (mg/dl)	268±101	267±122	0.98
TC (mg/dl )	240±51	233±50	0.27
HDL-C (mg/dl )	52±12	53±14	0.55
LDL-C (mg/dl )	119±28	114±29	0.13

Values are presented as mean±SD; *Significant differences between groups

**Table 2:** Genotype and allele frequencies of ADA gene SNP* G22A* in the GDM and non-GDM subjects

**Group**	**n**	**Genotype frequency**		**AA**	**P**		**Allele frequency**		**P **
		**GG**	**GA**				**G**	**A**	
Non GDM	70	59 (84%)	9 (13%)	2 (3%)	0.46		127 (91%)	13 (9%)	0.57
GDM	70	55 (79%)	14 (20%)	1 (1%)			124 (89%)	16 (11%)	
		OR (95% CI), 2.62 (1.08-6.36)					OR (95% CI), 1.26 (0.58-2.7)		

Genotypes are reported as number with percent in parentheses

**Table 3:** Frequencies of GG and GA+AA genotype in the GDM and non-GDM subjects

**Group**	**n**	**Genotype frequency**		**P**
		**GG**	**GA+AA**	
Non GDM	70	59 (84%)	11 (16%)	0.385
GDM	70	55 (79%)	15 (21%)	
		OR (95% CI), 1.46 (0.62-3.5)		

Genotypes are reported as number with percent in parentheses

**Table 4:** Demographic characteristics and biochemical measurements in the subjects with GG and GA+AA genotype in the GDM and Non-GDM group

	**GDM**		**Non-GDM **	
	**GG**	**GA+AA**	**GG**	**GA+AA**
N	55	15	59	11
Gestational age (week)	30.3±3.4	29.1±3.4	29.5±3.1	30.4±3.1
BMI (Kg/m^ 2 ^)	28.9±4.5	27.2±4.7	28.0±4.6	28.9±3.0
FPG (mg/ml)	90.4±23.6^#^	84.3±19.7	79.5±11.5	74.1±9.3
HbA1C (%)	6.0±1.0^#^	5.5±0.7	5.1±0.4	5.0±0.3
Insulin (µg/dl)	19.1±11.2	17.3±14.5	17.8±7.6	15.7±6.4
HOMA-IR	4.1±1.7	3.8±2.7	3.5±1.7	3.0±1.5
TG (mg/dl)	284±134	232±62	273±104	232±82
TC (mg/dl )	234±52	216±38	242±52	225±38
HDL-C (mg/dl )	52±13	50±13	53±13	49±6
LDL-C (mg/dl )	114±30	109±23	120±28	110±22

Values are presented as mean±SD; #Significant difference with non-GDM subjects (one way ANOVA, P<0.05).

## Discussion

In this study, the frequency of genotypes and alleles of SNP G22A were similar between GDM and non-GDM individuals. However, GDM patients with GG genotype had significantly poor glycemic control and higher frequency of obesity compared to patients with GA+AA genotypes. To the best of our knowledge, this study is the first that evaluates the G22A polymorphism of ADA in patients with GDM. In our study, the frequency of G/G; G/A; A/A genotypes and rare A allele among the non-diabetic women were closely comparable with that reported among the Caucasian population.^10^^,^^11^

The results revealed that HOMA-IR, FPG, and HbA1c were significantly higher in patients with GDM compared to healthy pregnant women. These findings suggest poor glycemic control and insulin resistance in GDM patients. The level of HOMA-IR did not differ between the patients with different genotypes; it suggests that SNP G22A in ADA gene is not associated with insulin resistance in this population. A significant increase in FPG and HbA1c has been observed in GDM patients with GG genotype compared to patients with GA+AA genotypes. Hence, it can be assumed that the GG genotype is associated with the impaired glucose metabolism in GDM. This finding is in accordance with previous studies, which demonstrated a positive association between the ADA activity and the level of HbA1c in T2D.^5^^,^^12^ Obesity is a major risk factor in the development of GDM.^13^ The results revealed a higher frequency of the GG genotype in obese GDM subjects compared to lean GDM subjects. In view of these findings, we can suggest that GG genotype is a risk factor for impaired glucose metabolism, poor glycemic control, and obesity in GDM. A possible mechanism for the association of GG genotype with these abnormalities is the higher enzymatic activity of ADA in individuals with the GG genotype.^7^ Increased ADA activity will reduce the level of adenosine, which abolishes the effects of adenosine on plasma glucose and weight loss.^14^

## Conclusion

Our study elucidates the association of GG genotype of SNP G22A with major clinical manifestations of GDM including impaired blood glucose homeostasis and obesity. However, GDM is a heterogeneous disease. Thus, the effect of a single genetic factor on the risk of GDM should be considered with other genetic or environmental risk factors. 
